# Supplementary material for: Live-cell p53 single-molecule binding is modulated by C-terminal acetylation and correlates with transcriptional activity
Source: Nat Commun. 2017 Aug 22;8:313. doi: 10.1038/s41467-017-00398-7 (PMC5567047; doi:10.1038/s41467-017-00398-7)
Supplement: Supplementary file 1 — Supplementary Information [file 41467_2017_398_MOESM1_ESM.pdf]

File Name: Supplementary Information
Descriptions: Supplementary Figures, Supplementary Table and Supplementary Methods

File Name: Supplementary Movie 1
Descriptions: Example of SMT acquisition on HaloTag-p53wt in MCF-7/6/Hp53 cells in basal conditions. Acquisition rate 10 fps displayed in real time. Shown are the raw movie, the maximum projection of the movie – blurred with a Gaussian filter (sigma = 2 pixels) and the superimposed tracks, generated using the TrackMate plug-in in Fiji 1.

File Name: Supplementary Movie 2
Descriptions: Example of SMT acquisition on HaloTag-p53wt in MCF-7/6/Hp53 cells 2 hrs after the induction of DNA damage by 10 Gy IR. Acquisition rate 10 fps, displayed in real time. Shown are the raw movie, the maximum projection of the movie – blurred with a Gaussian filter (sigma = 2 pixels) and the superimposed tracks, generated using the TrackMate plug-in in Fiji 1.

File Name: Supplementary Movie 3
Descriptions: Example of identified HaloTag-p53 bound molecules by the kymographs approach in MCF-7/6/Hp53. Acquisition rate 10 fps, displayed 30 fps. Identified bound molecules are highlighted with yellow marks.

File Name: Supplementary Movie 4
Descriptions: Example of SMT acquisition for HaloTag-p53wt in H1299 cells in basal conditions. Acquisition rate 25 fps, displayed in real time.

File Name: Supplementary Movie 5
Descriptions: Example of SMT acquisition for HaloTag-p53wt in H1299 cells 2 hours after the induction of DNA damage by 10 Gy IR. Acquisition rate 25 fps, displayed in real time.

File Name: Supplementary Movie 6
Descriptions: Example of SMT acquisition for HaloTag-p53mSB in H1299 cells in basal conditions. Acquisition rate 25 fps, displayed in real time.

File Name: Supplementary Movie 7
Descriptions: Example of SMT acquisition for HaloTag-p53mSB in H1299 cells 2 hours after the induction of DNA damage by 10Gy IR. Acquisition rate 25 fps, displayed in real time.

File Name: Peer Review File
Descriptions:

SUPPLEMENTARY METHODS.

A simplified method for extracting the bound fraction and the residence times from single molecule data.

We have previously described a thorough method to extract kinetic parameters of individual molecules binding to immobile substrates, based on the modelling and fitting of the spatiotemporal distribution of displacements[2] (that is the distribution of displacements for $t = \Delta t, t = 2\Delta t, \ldots t = n\Delta t$ with Δt being the time between two consecutive frames). While the previously described method provides robust estimates of TF binding, it is also very sensitive to tracking errors, since for long-lasting tracks even a few percent rate of mistracking can result in large underestimation of the residence time. For example if the localization/tracking algorithm has an error rate of 1% (corresponding to 1 lost displacement every 100), the duration of all binding events lasting longer than 100 frames will be underestimated.

In the original implementation of our SMT analysis pipeline, manual intervention was therefore necessary to correctly estimate the distribution of residence times. Here we devise a simplified analysis method that aims to quantify in two separate steps the fraction of bound molecules and their residence time, with no manual intervention. The bound fraction is calculated from analyzing the distribution of displacements at $t = \Delta t$, while the distribution of residence times are extracted by kymograph analysis, as detailed below. Since this simplified method is based on a number of assumptions, we also detail the control experiments to validate our analysis method.

Single molecule Tracking and analysis of histograms of displacements.

The collected SMT movies were analyzed with custom-written Matlab routines, to identify and track individual molecules, as previously described [2]. Resulting tracks were used to populate a histogram of single molecule displacements between consecutive frames with bin size Δr equal to 20 nm. The histogram was normalized to provide the probability $p(r)\Delta r$ of observing a molecule jumping a distance between $r - \Delta r/2$ and $r + \Delta r/2$ in the time between two consecutive frames Δt. Such probability was then fit by a three-component diffusion model [3,4]:

$$p(r)\Delta r = r\Delta r \sum_{i=1}^{3} \frac{f_i}{2(D_i\Delta t+\sigma^2)} \exp\left(-\frac{r^2}{4(D_i\Delta t + \sigma^2)}\right)$$

(Eq. S.1)

where f_i is the fraction of molecules with diffusion coefficient equal to D_i, so that $\sum_{i=1}^{3} f_i = 1$ and σ = 33nm is the accuracy with which individual molecules are localized, calculated by tracking single HaloTag-p53 molecules in the nucleus of formaldehyde fixed cells (Figure S2e). When comparing histograms of displacements obtained with different treatments, a global fitting with parameter sharing approach was used to obtain independent estimates of the fractions f_i and of the diffusion coefficients D_2 and D_3 for each of the tested conditions, while estimating global values for the diffusion coefficient of the immobile population D_1. To provide standard deviations on the fitting parameters a jackknife procedure was adopted as described in [5]. Briefly, we performed multiple fitting iterations, each of them after dropping 20% of the data for each of the data set. Errors are provided as standard deviations of the obtained distribution of parameters following 2000 individual fitting iterations.

A number of conditions need to be met to correctly provide an estimate of the bound fraction of this method.

(i) In order to be capable of resolving differently diffusing components, the frame rate needs to be low enough so that the different components are distinguishable from each other. The distribution of displacements of each of the component will have its maximum at $r_{\max(p)} = \sqrt{2(D\Delta t + \sigma^2)}$ and at $\Sigma = 2r_{\max(p)}$ the distribution will have dropped to 44% of its maximum. We therefore impose the frame rate so that the peak of the slowest free diffusing component will be found at larger r than Σ calculated for the immobile population $r_{\max(\text{mobile})} > \Sigma_{\text{Immobile}}$. For D_{mobile} = 0.2 μm^2/s and $\sigma_{\text{loc}} = 33$ nm, we find that if $\Delta t > 0.04$s, $r_{\max(\text{mobile})} > \Sigma_{\text{Immobile}}$. Throughout the work we therefore used frame-rates ranging from 25 fps to 10 fps.

(ii) A long time interval between consecutive frames might impact the estimation of the bound fraction, as displacements corresponding to free molecules might be underestimated since faster diffusing molecules have a larger probability of escaping the observation volume [6]. To correct the estimated fraction of molecules f_i, we performed Monte-Carlo simulations for diffusing molecules with coefficients equal to each of the measured D_i, as described in [2] in order to compute for each of the diffusing components the probability of remaining within the thickness of the observation slice (set to w =1.5 μm), Briefly, we run simulations for 1500 frames and 100 molecules diffusing in a cubic box of w_{box}= 10 μm edge and we counted the number of observed displacements, N_{obs} whose edges fall within the observation slice. A correction factor for each of the components f_i was then calculated as $N_{\text{theo}}/N_{\text{obs}}$, where N_{theo} is the number of

displacements one would have theoretically observed if the molecules initially in focus would have stayed in focus for the whole duration of the movie: $N_{\text{theo}} = (N_{\text{frames}} - 1) * w/w_{\text{box}} =$ 22485 displacements. The correction factors are then averaged on 10 independent runs of the simulation.

We verified the validity of our correction by applying it on simulated data with an imposed bound fraction equal to 50%, and a diffusion coefficient for the free molecules equal to 1.82 $\mu m^2/s$. The analysis of the distribution of displacements before correcting for the escape of free molecules provided a bound fraction equal to (58 ± 2)% that reduced to (49± 4)% following the correction. The validity of the correction was also tested by analyzing data acquired at a 10× faster acquisition rate (see below).

(iii) Fitting the distribution of displacements with independent diffusing components assumes that negligible exchange between free and bound molecules occurs in the interval between two consecutive frames. We have measured that on average p53 binding events last more than 3s. The probability of having a bound molecule to become unbound in less than 0.1s is therefore lower than $1 - e^{-0.1\text{s}/3\text{s}}$, < 4%. Further, these fast unbinding events will contribute to the distribution of displacements with one single jump, while on average bound molecules will contribute with >30 jumps each. Therefore, for our current experiments the effect of neglecting the exchange between free and bound molecules in consecutive frames will affect the estimation of the bound fraction for less than 0.2%.

(iv) Long interval time between consecutive frames can impact the correct tracking of molecules, as the track of diffusing molecules might overlap. Quantification of mistracking is non-trivial since it depends on the density of localized molecules which varies over time due to photobleaching. We therefore tested whether the estimated diffusion coefficients and bound fractions were affected by potential mistracking by acquiring data at a faster frame rate (100 fps) for a selected condition (namely 2 hours after the induction of DNA damage by IR). At these acquisition rates, it is not possible to separate the different populations of free molecules from just the distribution of displacements between consecutive frames (see point (i)), and we therefore analyzed these data by fitting the full spatiotemporal distribution of displacements following manual verification of the tracks, as described in [2]. The comparison of the results obtained at 10 and 100 fps frame rates are provided in Supplementary Figure 2c. Notably, the estimated bound fractions were found to perfectly overlap when comparing data obtained at the two different acquisition rates. However, a significant reduction of the diffusion coefficient for diffusing populations was observed. This observation confirms our previous SMT data on p53, where we observed that acquiring data at different frame rates impacts the estimation of the diffusion coefficient of the freely diffusing

molecules, but not the binding parameters. In [2] we assigned these differences in the estimation of the diffusion coefficient to the fact that, due to the crowded nature of the nuclear environment, p53 does not diffuse freely, but anomalously, so that the estimated diffusion coefficient depends on the time over which it is calculated according to the formula:

$$D(\Delta t) = \Gamma \Delta t^{\alpha-1}$$

(Eq. S.2)

Where $\alpha = 0.82$ for p53 in the nucleus of H1299 cells. Accordingly, we can calculate the apparent diffusion coefficient expected to be measured at $\Delta t = 100$ ms, based on the one measured at $\Delta t = 10$ ms as:

$$D_{100\text{ms}} = D_{10\text{ms}} * 0.1^{\alpha-1} = 1.85\ \mu\text{m}^2/\text{s},$$

(Eq. S.3)

In good agreement with the diffusion coefficient measured using a frame rate of 10 fps.

Nevertheless, we note that the lower diffusion coefficients measured at 10 fps might also be partially caused by increased chances of mistracking at the lower frame rate: this might lead to an overestimation of the bound fraction since free molecules could jump out of focus more frequently than accounted for by our correction (point ii above). To evaluate the maximum error that might affect our measured bound fraction caused by an underestimation of the diffusion coefficients, we simulated single molecule data at 10 fps by imposing the diffusion coefficient measured at 100 fps ($2.9 \mu\text{m}^2\text{s}^{-1}$), and by considering a detection slice w =1 µm (to account for an eventual overestimation of this parameter) and a bound fraction equal to 50%. We then fit the resulting histogram of displacements and corrected the estimates of the bound fraction by using the parameters used in the manuscript ($D = 1.8\ \mu\text{m}^2\text{s}^{-1}$, w =1.5 µm), resulting in an estimated bound fraction equal to $(60 \pm 5)\%$. In our experimental conditions we have measured bound fractions ranging between 10% and 25%. If mistracking is responsible for the reduced diffusion coefficient at 10fps, this error would therefore impact our estimated bound fractions by 2% to 6%.

Measurement of the duration of the binding events by kymograph analysis

Kymograph analysis is carried out using custom written Matlab routine, allowing to identify the kymograph segments corresponding to immobile molecules: movies are first bandpass filtered to sharpen the signal corresponding to single molecules. Binarized stacks are obtained by using different thresholds and binary objects lasting less than a defined threshold are discarded. For more robust detection of binding events two different thresholds are used (the first one - >150 AU

for at least 9 frames - to detect dimmer signals lasting for a longer time and the second one - >200 AU for at least 4 frames - to include brighter signals that might last shorter). The images resulting from this binarization are combined together by an OR operation, and morphological closing is applied to close eventual 1 time-point gaps in the traces. Finally, particles displacing more than +/- 3 pixels and lasting less than 10 frames are discarded from the analysis. The binding events detected in this fashion are then used to populate a cumulative histogram of duration of binding event, which is then inverted, normalized to the total number of detected binding events and photobleaching corrected as previously described [7], to generate the complement cumulative density function (1 –CDF) of the duration of binding events.

The requirement for the identified segments to be continuous, corresponds to select only molecules moving less than $r_{\mathrm{max}} = 290$ nm between consecutive frames.

Further, the choice of discarding identified segments of length $N_{\mathrm{min}} < 10$ is to minimize false positives contributed by free molecules transiently moving slow enough to be indistinguishable from immobilized ones. We can calculate the probability that a free molecule with a diffusion coefficient D_{f} will be erroneously counted as bound by:

$$P(r_{\mathrm{max}}, N_{\mathrm{min}}) = \left(1 - e^{-\frac{r_{\mathrm{max}}^2}{4D_{\mathrm{f}}\Delta t}}\right)^{N_{\mathrm{min}}}$$

(Eq. S.4)

And for the chosen thresholds, $P \leq 1\%$ for $D_{\mathrm{f}} \geq 0.2\ \mu\mathrm{m}^2/\mathrm{s}$. When tested on the subset of data displayed in Figure 1d, the distribution of residence times obtained by the kymograph analysis were found to overlap with the distribution obtained by tracking-based objective thresholding [2] – (Supplementary Figure S2d). To quantify residence times, the complement cumulative density function 1- CDF is then fit with a two-component exponential decay:

$$1 - CDF = F_{\mathrm{s}} \exp(-t/\tau_{\mathrm{s}}) + (1 - F_{\mathrm{s}}) \exp(-t/\tau_{\mathrm{ns}})$$

(Eq. S.5)

to provide estimates for the average residence time τ_{s} of the long-lived (specific) bound population, the average residence time τ_{ns} of the short-lived (non-specific) one, and the fraction of binding events observed in the long-lived population F_{s}. The average residence time is then calculated as $\overline{\tau} = F_{\mathrm{s}}\tau_{\mathrm{s}} + (1 - F_{\mathrm{s}})\ \tau_{\mathrm{ns}}$

Relationship between average residence time, frequency of specific binding events and bound fraction for a two-binding states model.

To obtain information about the free time between two binding events, we consider a two-binding states model described by the following set of first-order differential equations:

$$\frac{\mathrm{d}F}{\mathrm{d}t} = -k_{\mathrm{on_s}}F - k_{\mathrm{on_n}}F + k_{\mathrm{off_s}}S + k_{\mathrm{off_n}}N$$
$$\frac{\mathrm{d}S}{\mathrm{d}t} = +k_{\mathrm{on_s}}F - k_{\mathrm{off_s}}S$$
$$\frac{\mathrm{d}N}{\mathrm{d}t} = +k_{\mathrm{on_n}}F - k_{\mathrm{off_n}}N$$

(Eq. S.6)

Where F, S and N are respectively the concentrations of free, specifically bound and non-specifically bound molecules, $k_{\mathrm{off_s}}$and $k_{\mathrm{off_n}}$are the dissociation rates of the transcription factor to specific and non-specific binding sites (the inverse of τ_{s} and τ_{ns}) and $k_{\mathrm{on_s}}$and $k_{\mathrm{on_n}}$ are the corresponding pseudo-association rates (association rates multiplied by the concentration of available binding sites). We note that $k_{\mathrm{on_s}}$+$k_{\mathrm{on_n}}$is the association rate of a molecule to any binding site (either specific or non-specific) and therefore the average free time between two binding events is equal to $\tau_{3\mathrm{D}} = \frac{1}{k_{\mathrm{on_s}}+k_{\mathrm{on_n}}}$

We can calculate the probability F_{s} that an association event is an association to a specific site, by considering the reduced equation set – no dissociation considered:

$$\frac{\mathrm{d}F^*}{\mathrm{d}t} = -k_{\mathrm{on_s}}F^* - \mathrm{k}_{on_n}F^*$$
$$\frac{\mathrm{d}S^*}{\mathrm{d}t} = +k_{\mathrm{on_s}}F^*$$
$$\frac{\mathrm{d}N^*}{\mathrm{d}t} = +k_{\mathrm{on_n}}F^*$$

(Eq. S.7)

And by calculating:

$$F_{\mathrm{s}} = \frac{\frac{\mathrm{d}S^*}{\mathrm{d}t}}{\frac{\mathrm{d}S^*}{\mathrm{d}t} + \frac{\mathrm{d}N^*}{\mathrm{d}t}} = \frac{k_{\mathrm{on_s}}}{k_{\mathrm{on_s}} + k_{\mathrm{on_n}}}$$

(Eq. S.8)

The probability F_{s} is equal to the fraction of binding events measured in the long-living state by the 1-CDF distribution of residence times as we verified by Monte-Carlo simulations (data not shown).

We can now relate the measured bound fraction to F_S and τ_{3D} by considering the system at equilibrium:

$$-k_{\mathrm{on_s}}F - k_{\mathrm{on_n}}F + k_{\mathrm{off_s}}S + k_{\mathrm{off_n}}N = 0$$
$$+k_{\mathrm{on_s}}F - k_{\mathrm{off_s}}S = 0$$
$$+k_{\mathrm{on_n}}F - k_{\mathrm{off_n}}N = 0$$

(Eq. S.9)

And by calculating the quantity:

$$f_1 = \frac{S+N}{S+N+F} = \frac{\frac{k_{\mathrm{on_s}}}{k_{\mathrm{off_s}}} + \frac{k_{\mathrm{on_n}}}{k_{\mathrm{off_n}}}}{\frac{k_{\mathrm{on_s}}}{k_{\mathrm{off_s}}} + \frac{k_{\mathrm{on_n}}}{k_{\mathrm{off_n}}} + 1} = \frac{k_{\mathrm{off_n}}k_{\mathrm{on_s}} + k_{\mathrm{off_s}}k_{\mathrm{on_n}}}{k_{\mathrm{off_n}}k_{\mathrm{on_s}} + k_{\mathrm{off_s}}k_{\mathrm{on_n}} + k_{\mathrm{off_s}}k_{\mathrm{off_n}}}$$

(Eq. S.10)

Dividing up and down for $k_{on_s} + k_{on_n}$ and for $k_{off_s}k_{off_n}$

$$f_1 = \frac{\frac{F_{\mathrm{s}}}{k_{\mathrm{off_s}}} + \frac{1-F_{\mathrm{s}}}{k_{\mathrm{off_n}}}}{\frac{F_{\mathrm{s}}}{k_{\mathrm{off_s}}} + \frac{1-F_{\mathrm{s}}}{\mathrm{k_{off_n}}} + \frac{1}{k_{\mathrm{on_s}} + k_{\mathrm{on_n}}}}$$

(Eq. S.11)

We note that: $\frac{F_{\mathrm{s}}}{k_{\mathrm{off_s}}} + \frac{1-F_{\mathrm{s}}}{k_{\mathrm{off_n}}} = \bar{\tau}$ and that $\tau_{3D} = \frac{1}{k_{\mathrm{on_s}}+k_{\mathrm{on_n}}}$, leading to:

$$f_1 = \frac{\bar{\tau}}{\bar{\tau} + \tau_{\mathrm{3D}}}$$

(Eq. S.12)

The average free time between two binding events can therefore be calculated as

$$\tau_{\mathrm{3D}} = \bar{\tau}(1 - f_1)/f_1$$

(Eq. S.13)

The estimated parameters τ_{ns}, τ_{3D} and F_{s} can be combined to quantify the p53 *search time,* the average time spent by a p53 to find one of its specific binding sites, by a modification of the analysis performed in [8]. The search time can be obtained by knowing the number of binding events that a molecule needs to undergo on average before encountering a specific binding site, $N_{\mathrm{Trials}} = 1/F_{\mathrm{s}}$.

Any trial round will take a time equal to $\tau_{\mathrm{3D}} + \tau_{\mathrm{ns}}$, except for the last one, which will last τ_{3D}, after which a specific site is found. We can therefore calculate the search time as:

$$\tau_{\mathrm{search}} = N_{\mathrm{trials}}\tau_{3D} + (N_{\mathrm{trials}} - 1)\tau_{\mathrm{ns}}$$

(Eq. S.14)

SUPPLEMENTARY FIGURES.

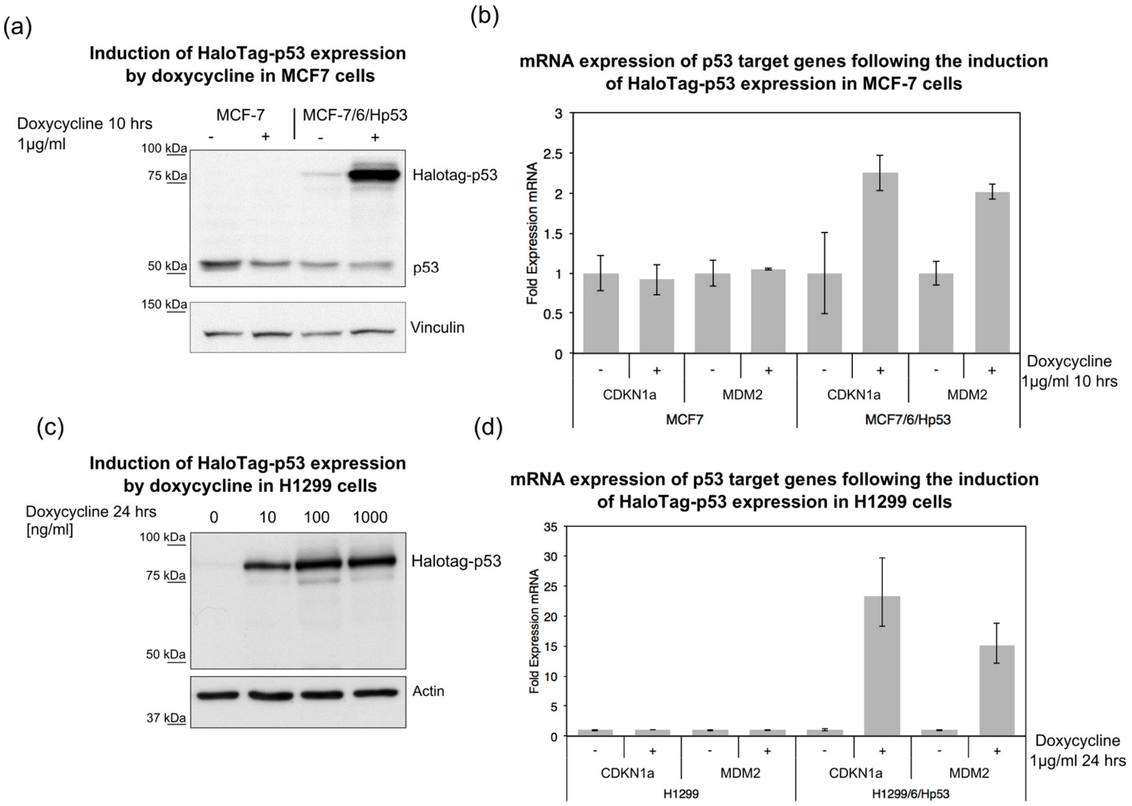

Supplementary Figure 1. ***Related to Figure 1.*** **Validation of MCF-7/6/Hp53 and H1299/6/Hp53 cell lines.** (**a**) Western blot characterization of HaloTag-p53 expression in MCF-7/6/Hp53 cells upon induction with Doxycycline for 10 hrs. (**b**) mRNA expression of p53 target genes by qPCR upon induction with doxycycline for 10 hrs (2 replicates, error bars: SD). (**c**) Western blot characterization of HaloTag-p53 expression in H1299/6/Hp53 cells upon induction with Doxycycline for 24 hrs. **(d)** mRNA expression of p53 target genes by qPCR upon induction with doxycycline for 24 hrs (2 replicates, errorbars: SD).

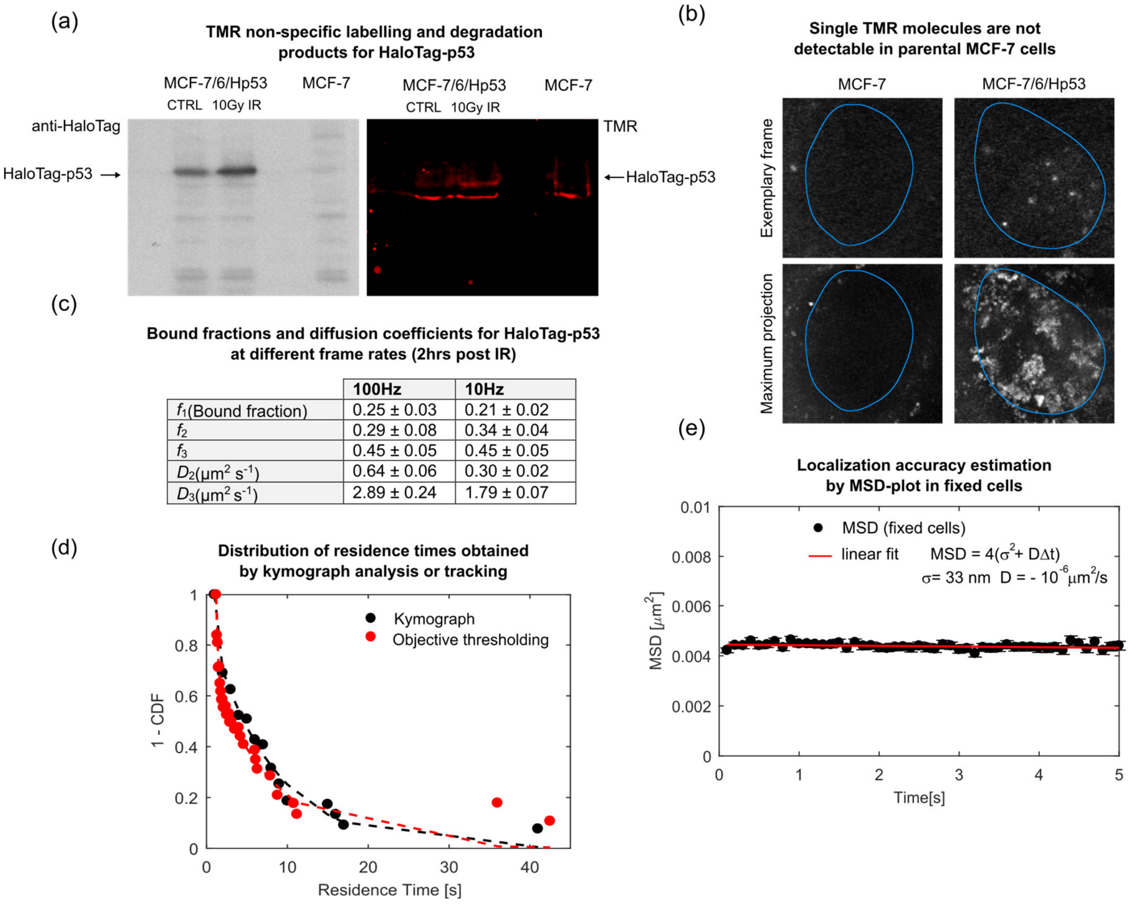

	100Hz	10Hz
f_1(Bound fraction)	0.25 ± 0.03	0.21 ± 0.02
f_2	0.29 ± 0.08	0.34 ± 0.04
f_3	0.45 ± 0.05	0.45 ± 0.05
D_2(μm² s⁻¹)	0.64 ± 0.06	0.30 ± 0.02
D_3(μm² s⁻¹)	2.89 ± 0.24	1.79 ± 0.07

Supplementary Figure 2. ***Related to Figure 1 and 2.*** **Validation of HaloTag-p53 single molecule imaging.** (**a**) Western blots on HaloTag-p53 in MCF-7 cells. We detected HaloTag-p53 using an anti-HaloTag antibody (left panel) and by direct visualization of the TMR ligand (right panel). In both cases, we could detect additional bands at lower molecular weight. These bands do not appear to be due to degradation products of HaloTag-p53, as they are visible also for the parental cell line MCF-7, but rather seem non-specific signals from the antibody and from the TMR ligand. (**b**) To exclude that such non-specific labelling could affect the single molecule tracking of HaloTag-p53 in the nucleus of living cells we labelled parental cell lines with TMR according to our standard protocol and verified that no single molecules could be identified and tracked in the nucleus of these cells. (**c**) Single molecule experiments obtained on MCF7/6/Hp53 cells provide comparable estimates for the bound fractions independently on the frame rate used. The diffusion coefficients for the free populations, instead, are found to increase when using faster frame rates. (**d**) The kymograph approach provides overlapping distributions of residence times to those found using a previously published method based on tracking[2]. (e) Estimation of the single-molecule localization accuracy of our experimental settings by tracking HaloTag-p53 in fixed cells.

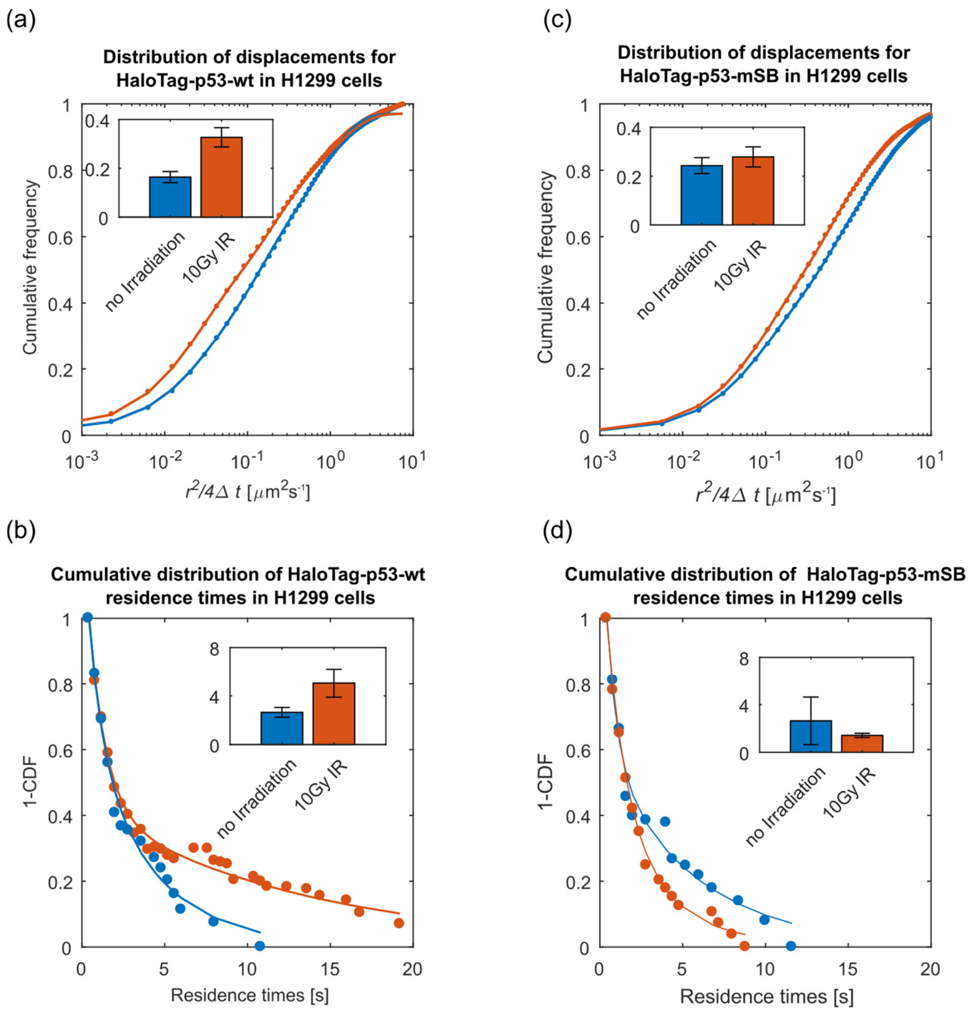

Supplementary Figure 3. ***Related to Figure 2*****. SMT analysis of HaloTag-p53-wt and HaloTag-p53-mSB in H1299 cells.** (**a**) Distribution of displacements for HaloTag-p53-wt in H1299 cells. Similarly to what observed for HaloTag-p53 in MCF7-6 cells, the HaloTag-p53 bound fraction increases upon irradiation with 10 Gy IR (n_{cells} = 6, 10 for no irradiation and 10 Gy IR respectively). (**b**) Kymograph analysis of the single molecule movies for HaloTag-p53-wt revealed that the average residence time of p53 on chromatin increases upon irradiation. Differently from wild type, neither the fraction of HaloTag-p53-mSB bound molecules (**c**) nor the HaloTag-p53-mSB average residence time on chromatin (**d**) increases following irradiation with 10 Gy IR (n_{cells} = 7, 6 for no irradiation and 10 Gy IR respectively, error bars: SD for bound fractions, 95% CI for average residence times).

(a)

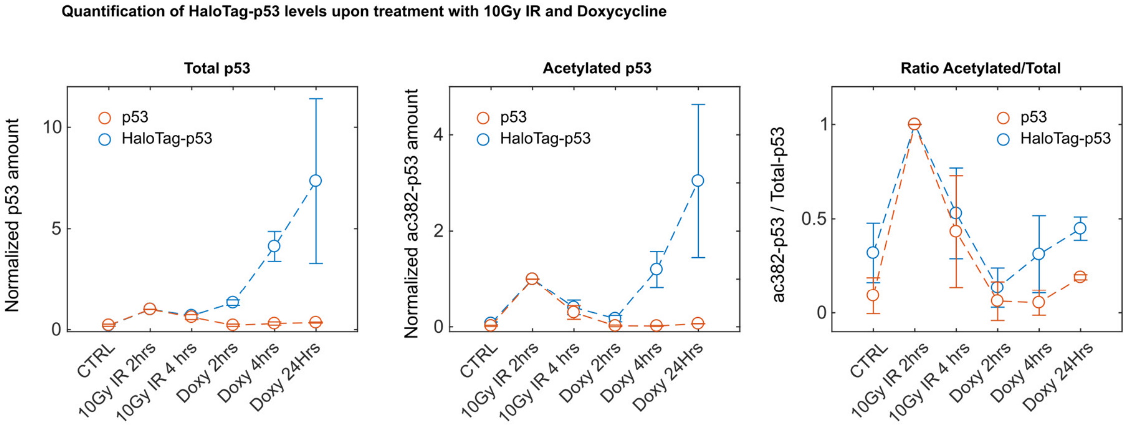

(b)

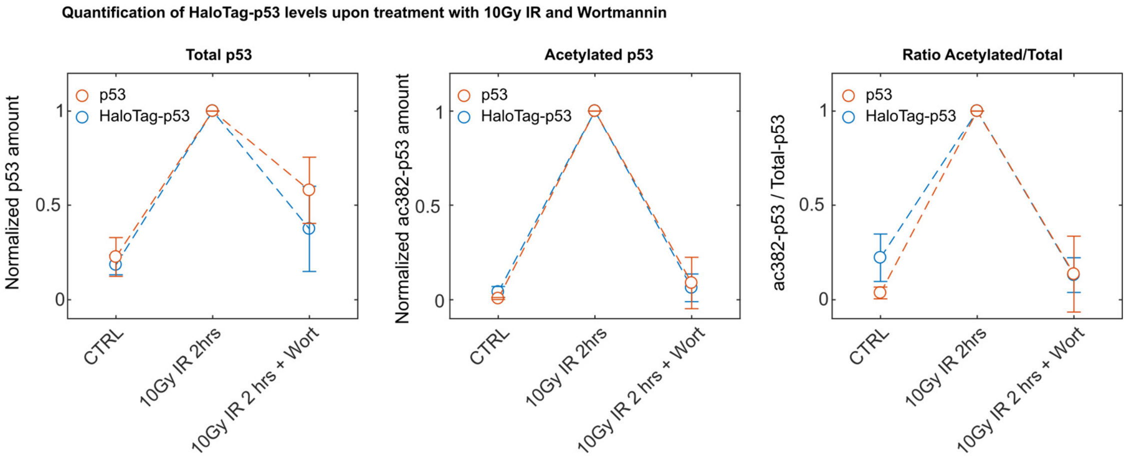

Supplementary Figure 4. ***Related to Figure 3*****. Quantification of HaloTag-p53 total levels and acetylation levels.** (**a**) The western blot of Figure 3a was repeated 3 times, and analyzed with ImageJ, by normalizing total (HaloTag)-p53 levels and (Halotag)-p53K382ac levels to Vinculin levels upon treatment with either 10 Gy IR or doxycycline for different times (3 replicates, error bars: SD). (**b**) The western blot of Figure 3d was repeated 4 times, and analyzed with ImageJ, by normalizing total (HaloTag)-p53 levels and (Halotag)-p53K382ac levels to Vinculin levels upon treatment with either 10 Gy IR or 10 Gy IR + wortmannin (4 replicates, error bars: SD).

(a)

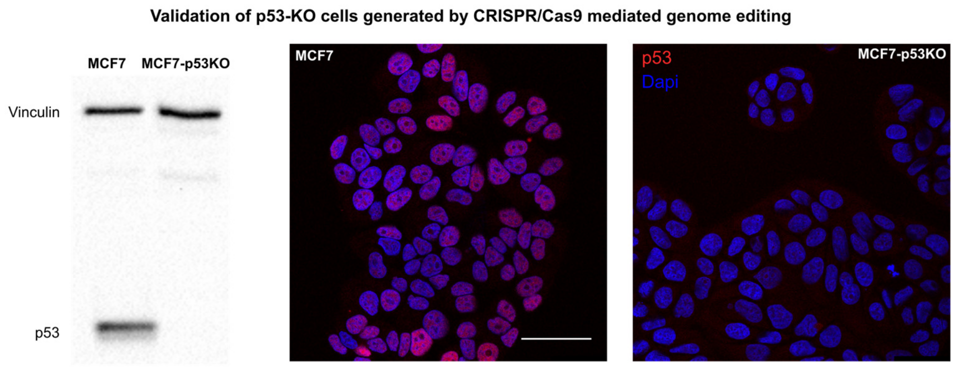

(b)

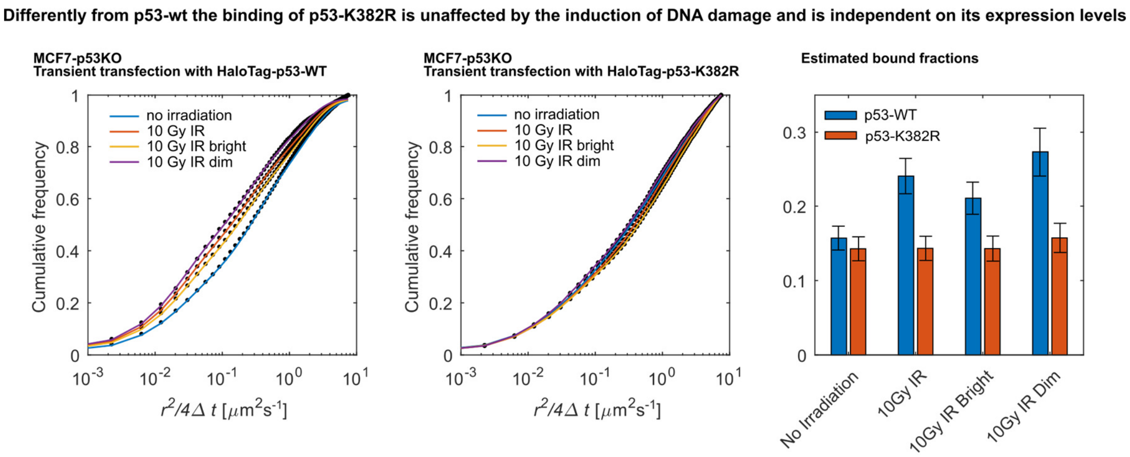

Supplementary Figure 5. ***Related to Figure 5.*** **Single molecule imaging experiments on MCF-7-p53KO cell lines. (a)** We generated MCF-7 cells knocked out for endogenous p53 (MCF7-p53KO) by CRISPR/Cas9 genome editing and we validated them by western blo t and immunofluorescence. Scale bar 50 µm. **(b)** We performed single molecule tracking on MCF7-p53KO cells transiently transfected with either HaloTag-p53-wt (left panel, two replicates, n_{cells} = 15, 17 for no irradiation and 10 Gy IR respectively) or HaloTag-p53-K382R (central panel, two replicates, n_{cells} = 17, 21 for no irradiation and 10Gy IR respectively). While p53-wt displays an increase in bound fraction following activation by 10 Gy IR, the K382R mutant does not show any modulation in binding (right panel, error bars: SD), comparable to what observed in H1299 cells. We also analyzed separately the higher-expressing 40% of HaloTag-p53 cells (10 Gy IR Bright) and the lower-expressing 40% of cells (10 Gy IR Dim). The measured bound fraction for cells highly expressing HaloTag-p53-wt is 6% lower than for low-expressing cells, while no dependence on the expression level is observed for the bound fraction of the HaloTag-p53-K382R mutant.

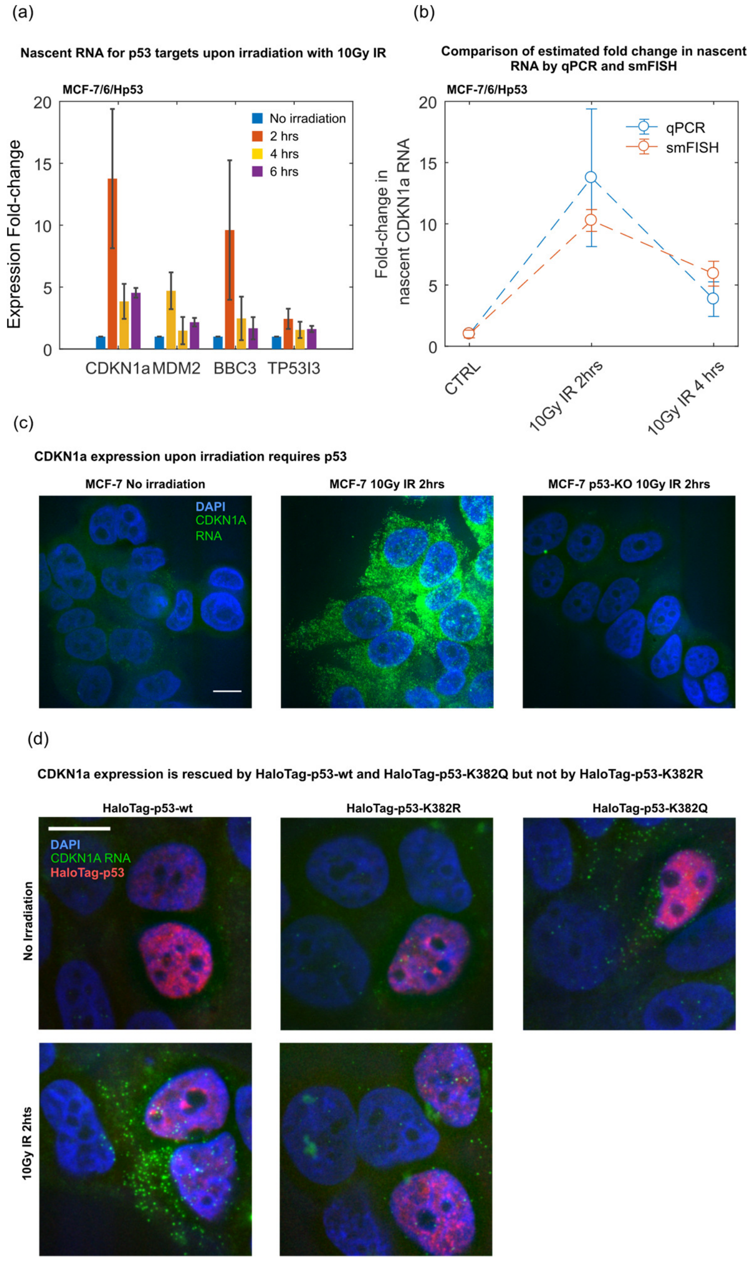

Supplementary Figure 6. ***Related to Figure 5*****. Nascent RNA by qPCR and smFISH** (**a**) Nascent RNA for p53 targets upon irradiation with 10Gy IR (3 replicates, error bars: SD). (**b**) Comparison of measured fold-change in CDKN1a nascent transcripts by qPCR and by smFISH (error bars: SD for qPCR, SEM for smFISH). (**c**) CDKN1a transcription depends on p53 as MCF-7 cells knocked out for endogenous p53 do not display smFISH signal. Scale bar 10 µm. (**d**) HaloTag-p53-wt transiently transfected in MCF-7-KO cells rescues CDKN1a transcription, while HaloTag-p53-K328R does not. Transient transfection of the acetylation mimicking mutant K328Q in MCF-7-KO cells results in detectable CDKN1a transcripts in basal conditions. Scale bar 10 µm.

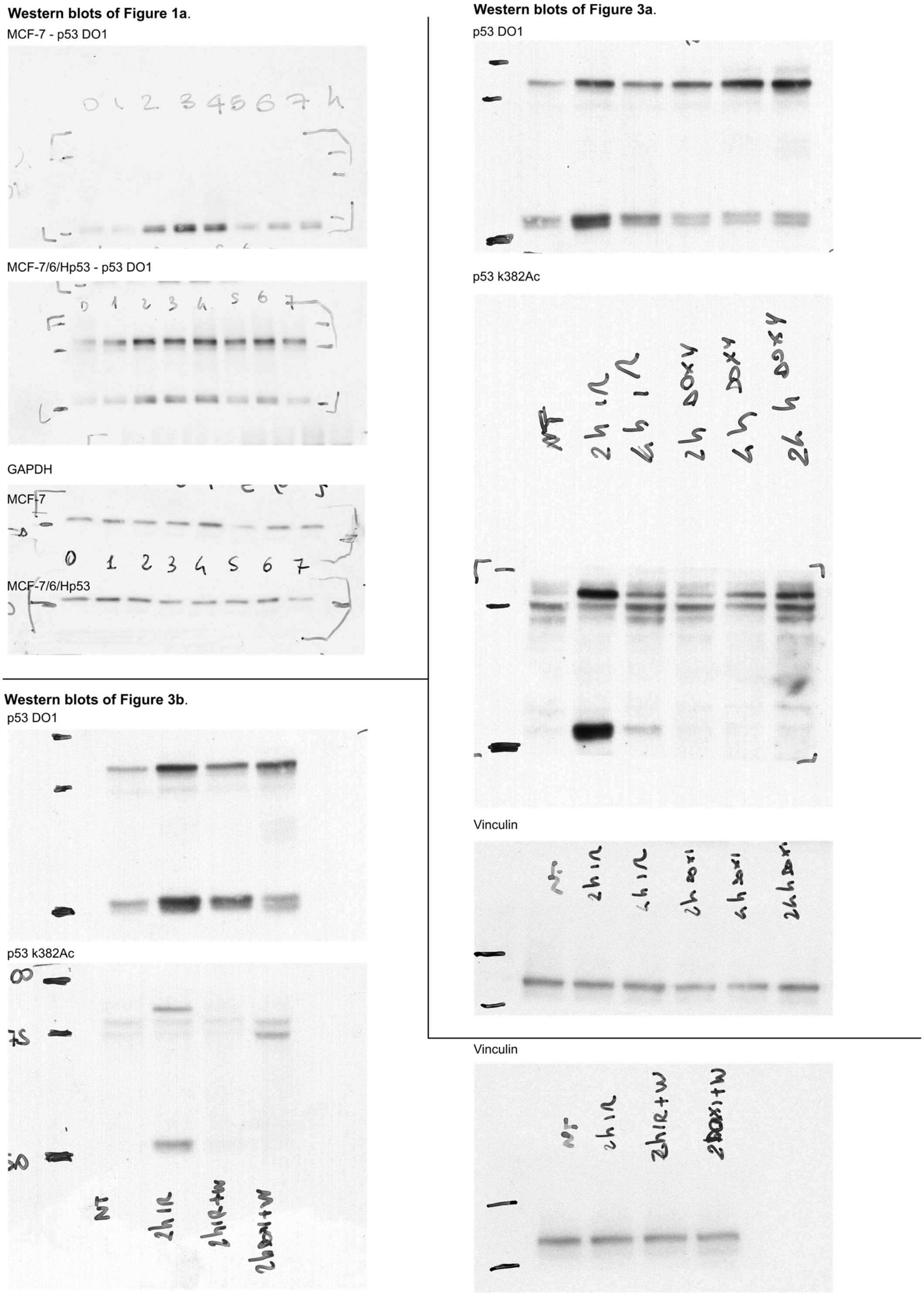

Supplementary Figure 7. ***Related to Figure 1 and Figure 3.*** Full scans of Western Blots shown in Figure 1 and Figure 3

Primers for mRNA targets (mature transcripts)	
CDKN1a (p21) Fw:	5'-CTGGAGACTCTCAGGGTCGAAA-3',
CDKN1a (p21) Rev:	5'-GATTAGGGCTTCCTCTTGGAGAA-3';
MDM2 Fw:	5'-GTGAATCTACAGGGACGCCATC-3'
MDM2 Rev:	5'-CTGATCCAACCAATCACCTGAA-3'
BBC3 (PUMA) Fw:	5'-GAAGAGCAAATGAGCCAAACG-3'
BBC3 (PUMA) Rev:	5'-GGAGCAACCGGCAAACG-3'
TP53I3 (PIG3) Fw:	5'-TCTCTGAAGCAACGCTGAAATTC-3'
TP53I3 (PIG3) Rev:	5'-ACGTTCTTCTCCCAGTAGGATCC-3'
GAPDH Fw:	5'-AGGGCTGCTTTTAACTCTGGT-3
GAPDH Rev:	5'- CCCCACTTGATTTTGGAGGGA-3'
**Primers for pre-mRNA targets (nascent transcripts)**	
CDKN1a (p21) Fw:	5'-ACCAGGGCCTTCCTTGTATC-3'
CDKN1a (p21) Rev:	5'- GCATGGGTTCTGACGGAC-3'
MDM2 Fw:	5'- CAGAGGCACAGGGATGAGTT-3'
MDM2 Rev:	5'-CCTGAATGTTCACTTACACCAGC-3'
BBC3 (PUMA) Fw:	5'- CCCACTCCCATCACCATACT-3'
BBC3 (PUMA) Rev:	5'- CTGTGGCCCCTGGGTAAG-3'
TP53I3 (PIG3) Fw:	5'- ATATGCCTGTGTGGGGAATG-3'
TP53I3 (PIG3) Rev:	5'- CAGTCCTGCATGGATTAGCAC-3'
GAPDH Fw:	5'- CATGCCTTCTTGCCTCTTGT-3'
GAPDH Rev:	5'- GTTGAGGTCAATGAAGGGGTC-3'

Supplementary Table 1. List of primers used for qPCR.

SUPPLEMENTARY REFERENCES.

1. Schindelin, J. *et al.* Fiji: an open-source platform for biological-image analysis. *Nat. Methods* **9,** 676–682 (2012).
2. Mazza, D., Abernathy, A., Golob, N., Morisaki, T. & McNally, J. G. A benchmark for chromatin binding measurements in live cells. *Nucleic Acids Res.* **40,** e119 (2012).
3. Grünwald, D., Spottke, B., Buschmann, V. & Kubitscheck, U. Intranuclear binding kinetics and mobility of single native U1 snRNP particles in living cells. *Mol. Biol. Cell* **17,** 5017–5027 (2006).
4. Speil, J. *et al.* Activated STAT1 Transcription Factors Conduct Distinct Saltatory Movements in the Cell Nucleus. *Biophys. J.* **101,** 2592–2600 (2011).
5. Gebhardt, J. C. M. *et al.* Single-molecule imaging of transcription factor binding to DNA in live mammalian cells. *Nat. Methods* **10,** 421–426 (2013).
6. Harkes, R., Keizer, V. I. P., Schaaf, M. J. M. & Schmidt, T. Depth-of-Focus Correction in Single-Molecule Data Allows Analysis of 3D Diffusion of the Glucocorticoid Receptor in the Nucleus. *PLOS ONE* **10,** e0141080 (2015).
7. Mazza, D., Ganguly, S. & McNally, J. Monitoring Dynamic Binding of Chromatin Proteins In Vivo by Single-Molecule Tracking. in *Imaging Gene Expression* (ed. Shav-Tal, Y.) 117–137 (Humana Press, 2013).
8. Chen, J. *et al.* Single-molecule Dynamics of Enhanceosome Assembly in Embryonic Stem Cells. *Cell* **156,** 1274–1285 (2014).
